# Long-term sustainability of improvements in antibiotic prescribing after implementation of a local guideline for the management of patients hospitalized with skin and soft tissue infection

**DOI:** 10.1017/ash.2025.10063

**Published:** 2025-09-16

**Authors:** Kira E. Frappa, Katherine C. Shihadeh, Margaret M. Cooper, Paul D. Paratore, Timothy C. Jenkins

**Affiliations:** 1 Department of Pharmacy, Children’s Hospital Colorado, Aurora, CO, USA; 2 Department of Pharmacy, Denver Health Medical Center, Denver, CO, USA; 3 Division of Infectious Diseases, Department of Medicine, Denver Health Medical Center, Denver, CO, USA; 4 Division of Infectious Diseases, Department of Medicine, University of Colorado School of Medicine, Aurora, CO, USA

## Abstract

A local guideline for the management of patients hospitalized with skin and soft tissue infections was implemented at an academic, safety-net hospital. Immediate reductions in use of broad-spectrum antibiotics and durations of therapy were sustained over the subsequent 12 years.

## Introduction

Skin and soft tissue infections (SSTIs) are among the most common infections treated in hospitals. Several studies have demonstrated use of overly broad-spectrum antibiotics or excessive durations of therapy highlighting opportunities for antibiotic stewardship.^1–3^ Implementation of local treatment guidelines is an evidence-based antibiotic stewardship intervention recommended by CDC and required by Joint Commission.^4,5^ However, it is unknown if local guidelines are associated with long-term improvements in antibiotic use.

At Denver Health, an institutional guideline for the management of patients hospitalized with SSTIs was developed in 2009. We previously reported that implementation of the guideline was associated with significant reductions in use of antibiotics with a broad spectrum of gram-negative activity and durations of therapy over a one-year period.^6^ The purpose of this study was to evaluate whether these prescribing changes were sustained over a prolonged period.

## Methods

### Study design

This was a retrospective study of patients hospitalized at Denver Health Medical Center with a diagnosis of cellulitis or cutaneous abscess. The study consisted of three periods: (1) before implementation of the SSTI guideline (January 1, 2007–December 31, 2007; preintervention); (2) one-year period during which the guideline was actively implemented (July 9, 2009–July 8, 2010; intervention); and (3) period when there was no active intervention but the guideline remained in place (July 1, 2016–August 31, 2022; maintenance). Patient-level data were not accessible from the end of the intervention period until 2016 when a new electronic health recorded was implemented at Denver Health. The study was reviewed and granted exempt status by the Colorado Multiple Institutional Review Board.

### Data sources

Previously published data from the preintervention and intervention periods were included.^6^ To generate the cohort of patients from the maintenance period, the same data collection methodology was utilized. In brief, all patients age 18 years or older hospitalized with cellulitis or cutaneous abscess between July 1, 2016 and August 31, 2022 were identified by *International Classification of Diseases, 10^th^ Revision* (ICD-10) code search. A random subset of cases was reviewed manually to evaluate for eligibility. Those with complicated infections such as deep tissue infection, bacteremia, and recurrent infections were excluded. For eligible patients, demographics, select comorbid conditions, antibiotic use, and clinical outcomes were collected via a combination of electronic abstraction and medical record review using a standardized collection sheet. As this was quality improvement work, the sample size was dictated by the time available for data collection.

### Outcomes and statistical analysis

The co-primary outcomes were the proportion of patients exposed to antibiotics with a broad spectrum of gram-negative activity (β-lactam/β-lactamase inhibitors, fluoroquinolones, ceftriaxone, cefepime, or a carbapenem) and the total duration of antibiotic therapy.^6^ Secondary clinical outcomes included readmission within 30 days of discharge, in-hospital mortality, and hospital length of stay. Trends in the co-primary outcomes were analyzed by interrupted time series analysis (ITS) between periods using segmented regression models (autoregressive moving average (ARMA)[2,1] for antibiotics with broad gram-negative activity and ARMA[2,2] for duration of therapy). Aggregated data for the co-primary outcomes in each of the three periods were compared using the Kruskal-Wallis test of independent samples. R was used for development of the regression model and ITS analysis.^7,8^

## Results

The previously described preintervention and intervention period cohorts consisted of 169 and 175 cases, respectively. For the maintenance period, 4,898 cases were identified. Of these, 837 met at least one exclusion criterion by electronic data abstraction. Of the remaining 4,061, 486 random cases were manually reviewed; 300 met at least one exclusion criterion. In total, 186 cases during the maintenance period were included for analysis. Patient demographics, baseline comorbidities, and severity of illness were similar between the three periods (Table 1).

**Table 1.** Demographic and clinical characteristics

	Preintervention period (*N* = 169)	Intervention period (*N* = 175)	Maintenance period (*N* = 186)
**Age (years), median (IQR)**	46 (36–52)	47 (37–54)	45 (31–57)
**Sex, n (%)**			
Male	126 (75)	115 (66)	137 (74)
**Comorbidities, n (%)**			
Injection drug use	49 (29)	42 (24)	51 (27)
Diabetes mellitus	26 (15)	35 (20)	26 (14)
HIV infection	9 (5)	8 (5)	10 (5)
Cirrhosis	1 (1)	6 (3)	7 (4)
Alcohol use	27 (16)	22 (13)	42 (23)
Prior skin and soft tissue infection	23 (14)	29 (17)	37 (20)
Prior MRSA infection or colonization	11 (7)	7 (4)	5 (3)
**Clinical information, n (%) unless otherwise noted**			
Fever (temperature ≥ 38.0°C)	18 (11)	20 (11)	34 (18)
Leukocytosis (white blood cells > 10,000/mm^3^)	105 (62)	99 (57)	92 (49)
C-reactive protein (mg/L), median (IQR)^a^	50.1 (23.6–91.9)	62.5 (17.0–120.0)	68 (28.5–112.8)

Abbreviations: IQR, interquartile range; HIV, human immunodeficiency virus; MRSA, methicillin-resistant *Staphylococcus aureus,*

^a^Normal range, 0–10 mg/L.

**Figure 1.**
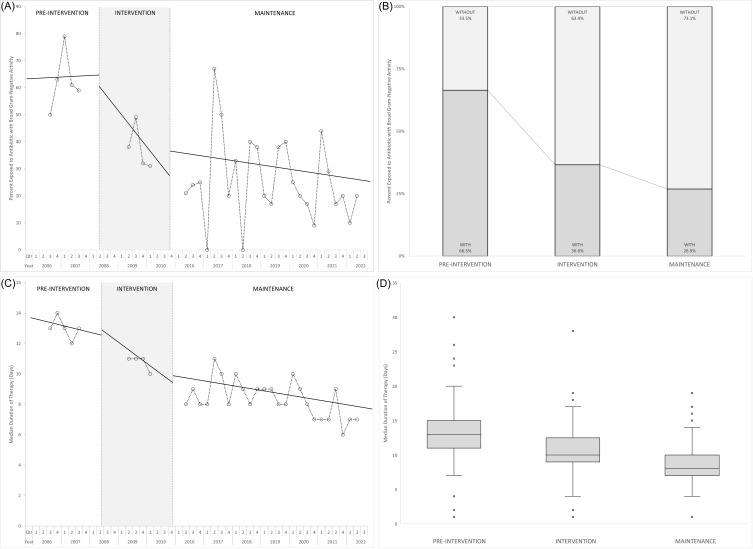
Panel A: Proportion of patients exposed to an antibiotic with broad gram-negative activity during the study, by quarter. Panel B: Aggregate proportion of patients exposed to an antibiotic with broad gram-negative activity during each study period. Panel C: Median total duration of antibiotic therapy during the study, by quarter. Panel D: Median total duration of antibiotic therapy during each study period (horizontal lines) with interquartile range (boxes), standard error (whiskers), and outliers (dots).

In the ITS model for exposure to broad-spectrum antibiotics, there was a non-significant immediate reduction in the proportion of patients exposed to an antibiotic with broad gram-negative activity between the preintervention and intervention periods (−14.3%, *P* = .55). Subsequently, there were no significant changes in trends over the intervention and maintenance periods; however, the degree of level change was maintained (Figure 1A). The aggregate proportion of patients exposed to an antibiotic with broad gram-negative activity was 67% during the preintervention period, 37% during the intervention period, and 27% during the maintenance period (*P* < .001) (Figure 1B). Specifically, exposure to agents with anti-pseudomonal activity occurred in 28%, 18%, and 16%, respectively (*P* < .001). In the ITS model for duration of therapy, there was a non-significant immediate reduction in duration of therapy between the preintervention and intervention periods (–1.6 d, *P* = .19). Subsequently, there were no significant changes in trends over the intervention and maintenance periods; however, the degree of level change was maintained (Figure 1C). The aggregate median total duration of therapy was 13, 10, and 8 days in the preintervention, intervention, and maintenance periods, respectively (*P* < .001) (Figure 1D). There were no notable differences between the three periods in 30-day hospital readmission, in-hospital mortality, and length of hospital stay (Supplemental Table 1).

## Discussion

Numerous studies have shown that implementation of local treatment guidelines for common infections improves antibiotic use.^6,9–10^ To our knowledge, this is the first study to evaluate the long-term effects of local guideline implementation on antibiotic use. Our findings have several important implications. First, they affirm the importance of local treatment guidelines for their effectiveness in improving antibiotic use. Second, unlike active interventions such as handshake stewardship or prospective audit and feedback, this intervention required few resources and did not require ongoing personnel time. This suggests that, particularly in settings with limited resources, the development of institutional guidelines for common infections should be prioritized. Finally, our data demonstrate that guidelines can result in lasting changes in prescribing behavior—more than 12 years in this example—suggesting they may contribute to true culture change.

Importantly, we observed that the discontinuation of the active components of our intervention (peer champion engagement, education, and department-level feedback) did not result in reversion to preintervention prescribing practices. In fact, additional reductions in use of broad-spectrum agents and duration of therapy were seen over time. This provides valuable insight that once buy-in from clinicians is achieved, desired prescribing patterns may be perpetuated without ongoing active intervention.

This study has several important limitations. First, given the retrospective, single-center design, the findings may not be generalizable. Additionally, it is unclear if the findings are applicable to other infections. Second, there was a 6-year gap (2010–2016) in our access to data immediately after the intervention period due to a change in Denver Health’s electronic health record; however, the 6 years of data during the maintenance period to which we did have access (2016–2022) showed consistent trends extrapolating from the intervention period. Finally, the number of included cases per quarter during the maintenance period was small. Although these small numbers led to substantial variability in the main outcomes, the trends over time were clear.

In summary, we demonstrated that after implementation of an institutional guideline for the management of patients hospitalized with cellulitis or abscess, observed reductions in use of broad-spectrum antibiotics and durations of therapy were sustained over the subsequent 12 years without active interventions targeting SSTIs. These findings suggest that evidence-based, local treatment guidelines can change the underlying prescribing culture within hospitals long-term and should be among the initial interventions implemented by antibiotic stewardship programs.

## Supporting information

10.1017/ash.2025.10063.sm001 Frappa et al. supplementary material Frappa et al. supplementary material

## Supplementary material

The supplementary material for this article can be found at https://doi.org/10.1017/ash.2025.10063
