# Supplementary material for: Long-term sustainability of improvements in antibiotic prescribing after implementation of a local guideline for the management of patients hospitalized with skin and soft tissue infection
Source: Antimicrob Steward Healthc Epidemiol. 2025 Sep 16;5(1):e216. doi: 10.1017/ash.2025.10063 (PMC12451805; doi:10.1017/ash.2025.10063)
Supplement: Frappa et al. supplementary material [file S2732494X25100636sup001.docx]

| **Supplemental Table 1.** Clinical outcomes | | | |
| --- | --- | --- | --- |
|  | **Pre-intervention period**  **(N = 169)** | **Intervention period**  **(N = 175)** | **Maintenance period**  **(N = 186)** |
| **Hospital readmission within 30 days, n (%)** | 13 (7.7) | 9 (5.1) | 15 (8.1) |
| **In-hospital deaths, n (%)** | 0 | 0 | 0 |
| **Length of hospital stay, median (interquartile range) days** | 4 (3-5) | 4 (3-5) | 3 (2-5) |
